# A three-dimensional measurement study of fracture displacement in Garden I femoral neck fracture: a retrospective study

**DOI:** 10.1186/s12891-023-06737-8

**Published:** 2023-08-01

**Authors:** Ying Wang, Jianxiong Ma, Haohao Bai, Hongqi Zhan, Bin Lu, Lei Sun, Hongzhen Jin, Xingwen Zhao, Yanfei Wu, Jiahui Chen, Xinlong Ma

**Affiliations:** 1 Tianjin Hospital, Tianjin University, Tianjin, 300050 China; 2 Clinical College of Orthopedics, Tianjin Medical University, Tianjin, 300070 China; 3 Tianjin Key Laboratory of Orthopedic Biomechanics and Medical Engineering, Tianjin, 300050 China

**Keywords:** Femoral neck fracture, Garden I fractures, 3D reconstruction, Displacement

## Abstract

**Background:**

Garden I femoral neck fractures are nondisplaced femoral neck fractures. Nonoperative treatment and in situ fixation are the preferred treatments. However, the postoperative outcome is not satisfactory and the incidence of complications remains high, which raises doubts about the accuracy of the diagnosis of nondisplaced Garden I fractures. Recently, three-dimensional (3D) reconstruction has been reported as a mature technology for reconstructing the bone structure of patients. We further extended this technique in the measurement of the fracture spatial displacement to verify the accuracy of Garden I femoral neck fractures.

**Methods:**

This was a retrospective study of patients with Garden I femoral neck fractures from January 2013 to December 2018 at our institution, who were included according to specified criteria. A bilateral proximal femur model of each patient was established based on computed tomography (CT) data. The displacement of the deepest portion of the femoral head fovea, the displacement of the center of the femoral head and the rotation of the femoral head were measured in the bilateral model.

**Results:**

A total of 102 patients diagnosed with Garden I fractures were included in this study. The cohort included 32 men and 70 women, with an average age of 55.88 ± 15.32 years. In these patients, the average displacement of the deepest portion of the femoral head fovea was 16.43 ± 7.69 mm. The minimum and maximum displacement was 3.58 and 44.32 mm, respectively. The average displacement of the center of the femoral head was 10.39 ± 5.47 mm and ranged from 2.16 to 34.42 mm. The rotational angle was 23.81 ± 10.15 ° and ranged from 3.71 ° to 61.19 °.

**Conclusions:**

Garden I fractures have large spatial displacement and cannot be considered incomplete or nondisplaced fractures. Therefore, we suggest that anatomical reduction should be considered during treatment.

## Background

Patients with femoral neck fractures are at substantial risk for disability, death and reduced quality of life [1–3]. Garden I fractures are called nondisplaced fractures, accounting for 15–33% of all femoral neck fractures [4, 5]. Despite the high frequency of the injury [6], no consensus exists about the optimal management of Garden I femoral neck fractures. Options include nonsurgical treatment, which involves fixation with plaster after traction or manual reduction; surgical treatment, which involves treatment with in situ fixation using multiple parallel cannulated screws and arthroplasty. Advocates of nonsurgical treatment perceive benefits with regard to patients’ prognostic function and quality of life compared with surgical treatment for Garden I fractures [7]. There are concerns, however, that nonsurgical treatment has a higher complication rate than surgical treatment and may increase the risk of secondary displacement [8, 9]. Primary arthroplasty may be considered for Garden I and II femoral neck fractures with posterior tilt ≥ 20°, especially among older patients [10].

Surgical treatment was also reported to be optimal [11]. Meta-analyses of studies involving patients with nondisplaced femoral neck fractures have suggested that surgical treatment results in a higher cure rate and lower rate of femoral head necrosis than nonsurgical treatment [12]. In the surgical treatment of nondisplaced femoral neck fractures, in situ fixation using multiple parallel cannulated screws has been favored by orthopedic surgeons [13, 14]. However, many previous studies report a high incidence of femoral head necrosis after internal fixation using various parallel implants for the surgical treatment of Garden I or II fractures [15–17].

Necrosis is caused by displacement of the femoral head, which results in the femoral head being subjected to improper forces [18]. The high incidence of osteonecrosis of the femoral head raises doubts about the accuracy of the diagnosis of nondisplaced Garden I fractures. The purpose of the current study was to precisely measure the spatial displacement of the femoral head in Garden I femoral neck fractures by using three-dimensional (3D) reconstruction and digital technology. These data were used to reevaluate the deficiency of Garden I fractures and to improve the diagnosis and treatment of these fractures in clinical practice.

## Materials and methods

### Study population

This retrospective study collected patients with Garden I femoral neck fractures from January 2013 to December 2018 at our institution. The fractures were classified based on the original Garden classification using the original preoperative anteroposterior radiographs [19]. All patients were selected with a standard diagnostic code, exclusion rule, and inclusion rule.

Inclusion criteria were as follows: (1) simple unilateral femoral neck fracture, and (2) fresh fracture. The exclusion criteria were as follows: (1) ipsilateral femoral shaft fracture or bilateral femoral neck fractures, (2) pathological femoral neck fracture, (3) congenital malformation, (4) previous femoral neck fracture, and (5) lack of preoperative anteroposterior radiographs or CT data.

This study was approved by our institutional ethics committee, and patients provided informed consent.

### Establishment of Model

The CT data of the patients were imported into Mimics 22.0 (Materialize, Leuven, Belgium) in DICOM format, which generated 3D models of the bilateral proximal femurs. Using the mirror function in Mimics, a mirror 3D model of the normal femur was established. Then, the mirror model of the normal femur was superimposed on the model of the fractured femur. Registration of two 3D models was completed according to the overlap of the greater and lesser trochanter and the direction of the femoral shaft.

### Three-dimensional measurement

**Fig. 1:**
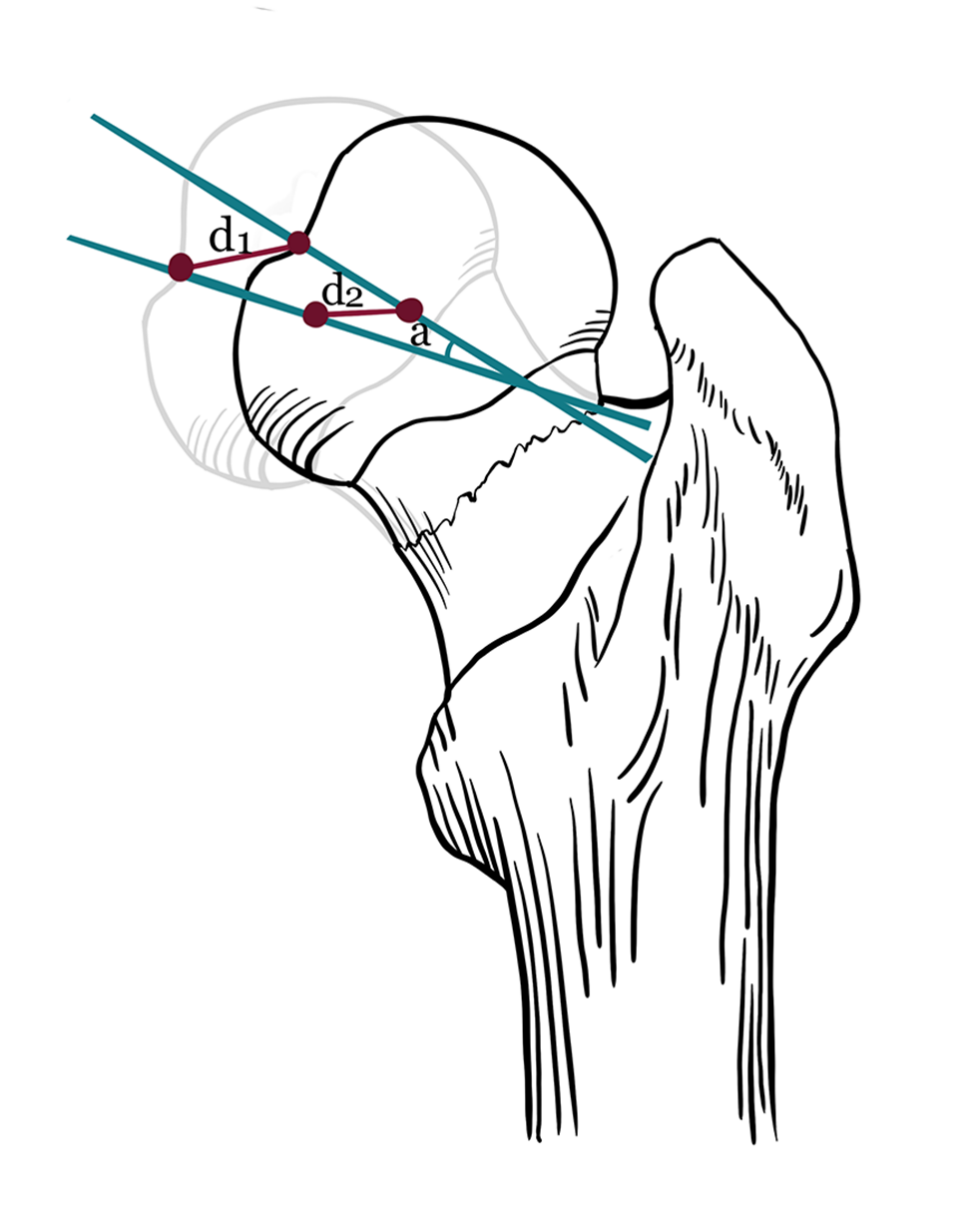
Schematic diagram of spatial displacement measurement method for femoral neck fractures

To measure the displacement and rotation of the femoral neck fracture, two distinct and constant anatomical landmarks were selected: the femoral head fovea and the femoral head center. The lowermost point was marked in the femoral head fovea. The femoral head center was calculated and marked automatically by selecting four points on the edge of the femoral head using a 3-matic module. The distance between the lowermost points of the bilateral femoral head fovea (d1) and the distance between the centers of the bilateral femoral heads (d2) were selected to describe the displacement of the femoral neck fracture. In addition, the angle between lines drawn between two feature points (α) was used to measure the rotation of the femoral neck fracture (Fig. 1).

### Statistical method

All data were analyzed using SPSS version 21 (IBM, USA). Means and standard deviations for the variables were obtained.

## Results

A total of 267 patients with femoral neck fractures in the Tianjin Hospital database during our study dates were diagnosed with Garden I fracture. Of these, 102 patients met our selection criteria. This cohort included 32 men and 70 women, with an average age of 55.88 ± 15.32.

In these patients, the average displacement of the deepest portion of the femoral head fovea was 16.43 ± 7.69 mm, the average displacement of the center of the femoral head was 10.39 ± 5.47 mm, and the rotational angle was 23.81 ± 10.15° (Table 1).

**Table 1:** Spatial Displacement of Garden I Femoral Neck Fractures

	N	Max	Min	Mean	SD
d1 (mm)	102	44.32	3.58	16.43	7.69
d2 (mm)	102	34.42	2.16	10.39	5.47
α (°)	102	61.19	3.71	23.81	10.15

## Discussion

Accurate diagnosis of fracture displacement is helpful in choosing the treatment method. Garden classification is commonly used in the clinic and is superior to other classification methods [20]. However, an increasing number of scholars have recently questioned the Garden classification. Chen [21]found that incomplete femoral neck fractures observed on radiographs are complete fractures on CT. Du’s three-dimensional reconstruction revealed a spatial displacement angle of 17.17 ± 10.40° for the inserted fracture, indicating the limitations of the Garden classification [22]. The results (Table 1) showed that the distance of the bilateral femoral head foveae was 3.58–44.32 mm, the distance of the bilateral femoral heads was 2.16–34.42 mm, and the rotation of the femoral head was 3.71–61.19°, which further confirms that the Garden classification is inaccurate in diagnosing fracture displacement.

Studies have found that the rate of femoral head necrosis, fracture redisplacement rate, and fracture nonunion rate are higher after nonsurgical treatment of Garden I fracture [23]. Based on the results of this study, we believe that patients with Garden I fractures cannot achieve effective reduction of fractures either conservatively or with in situ fixation surgery [24, 25]. However, it easily leads to fracture deformity and healing, increasing the risk of osteonecrosis of the femoral head. Therefore, we suggest that anatomic reduction is recommended for X-ray diagnosis of Garden I femoral neck fracture.

This study follows the measurement method proposed by Ma’s team [22]. Displacement measurement is based on bilateral limb symmetry and high reliability [18]. The feature points selected in the measurement are easy to identify on the femur and have a low error rate. Zhang [26]believes that this method has certain advantages in assessing fracture displacement. The limitation of this study is that the sample size is small. The next step is to continue the study using big data from multiple centers.

## Conclusions

Garden I femoral neck fractures have large spatial displacement and cannot be considered incomplete or nondisplaced fractures. Therefore, we suggest that CT and 3D reconstruction should be used in the diagnosis of femoral neck fractures to improve the accuracy of diagnosis and treatment, especially before selecting surgical options.

## Data Availability

The datasets used and analysed during the current study are available from the corresponding author on reasonable request.

## Abbreviations

3D: Three-dimensional
CT: Computed tomography
